# Neuropsychiatric Systemic Lupus Erythematosus Involvement: Towards a Tailored Approach to Our Patients?

**DOI:** 10.5041/RMMJ.10276

**Published:** 2017-01-30

**Authors:** Raquel Faria, João Gonçalves, Rita Dias

**Affiliations:** 1 Clinical Immunology Unit, Centro Hospitalar do Porto, Porto, Portugal; 2 Medical Service, Centro Hospitalar do Porto, Porto, Portugal

**Keywords:** Attribution models, functional magnetic resonance imaging, neuropsychiatric systemic lupus erythematosus

## Abstract

Neuropsychiatric involvement in systemic lupus erythematosus (NPSLE) is a complex condition that remains poorly understood, and includes heterogeneous manifestations involving both the central and peripheral nervous system, with disabling effects. There are several models to improve NPSLE diagnosis when a neurological syndrome is present. In the last couple of years, the growing knowledge of the role of cytokines and antibodies in NPSLE, as well as the development of new functional imaging techniques, has brought some insights into the physiopathology of the disease, but their validation for clinical use remains undetermined. Furthermore, besides the classic clinical approach, a new tool for screening the 19 NPSLE syndromes has also been developed. Regarding NPSLE therapeutics, there is still no evidence-based treatment approach, but some data support the safety of biological medication when classic treatment fails. Despite the tendency to reclassify SLE patients in clinical and immunological subsets, we hope that these data will inspire medical professionals to approach NPSLE in a manner more tailored to the individual patient.

## INTRODUCTION

Neuropsychiatric involvement in systemic lupus erythematosus (SLE) was first described by Moriz Kaposi in 1872 in a young woman with disturbed neurological function, among other systemic manifestations. In 1903, Sir William Osler raised the hypothesis that cerebral vascular changes could be responsible for neurological involvement in SLE.

Neuropsychiatric SLE (NPSLE) is currently described as “the most clinical challenging ‘visceral’ involvement,” “responsible for high morbidity and mortality,” and consequently posing a “great economic and social burden.”^1^

The condition has a broad spectrum of clinical manifestations and severity. The prevalence of NPSLE depends on which syndrome is present, and it is sometimes different from the prevalence of the same syndrome in the general population. In 1999 the American College of Rheumatology (ACR) proposed the most used and accepted nomenclature for NPSLE syndromes^2^ based on the previous classification attempts of Kassan et al.^3^ and Singer et al.^4^ They defined 19 different syndromes, distinguishing diffuse psychiatric/neuropsychological, and central and peripheral nervous system involvement,^2^ that could occur simultaneously or overlap in the same patient (Table 1, column one).

**Table 1:** Frequency of NPSLE Syndromes by ACR Nomenclature in Different Cohorts, *n* (%).

ACR Nomenclature	Ainiala, 2001^7^ (*n*=46)	Sanna, 2003^8^ (*n*=323)	Brey, 2002^9^ (*n*=128)	Afeltra, 2003^10^ (*n*=61)	Costallat, 2001^11^ (*n*=527)	Hanly, 2010^12^ (*n*=1206)		Mok, 2006^13^ (*n*=282)	Ferrara University, 2014^15^ (*n*=228)	Multiple Italian Centers, 2014^15^ (*n*=221)	Faria, 2014^16^ (*n*=55)	Steup-Beekman, 2013^14^ (*n*=102)

						Model A	Model B					

Aseptic Meningitis	1 (2)				2 (0.4)	4 (2.7)	4 (1.6)	1 (0.4)	3 (0.7)	0	6 (10.9)	3 (3)

Cerebrovascular Disease	7 (15)	47 (14.5)	2 (2)	15 (24)	13 (2.5)	18 (12.1)	40 (15.5)	21 (7.4)	68 (16.2)	61 (14.3)	16 (29)	44 (43)

Demyelinating Syndrome	1 (2)	3 (0.9)			1 (0.2)	1 (0.7)	3 (1.2)	0	3 (0.7)	4 (0.9)	1 (1.8)	

Headache	25 (54)	78 (24)	73 (57)	13 (21)	25 (62.5) (*n*=40)	0	0	8 (2.8)	116 (27.7)	95 (22.2)	4 (7.2)	23 (23)

Movement Disorder (Chorea)	1(2)	4 (1.2)	1 (1)		4 (0.76)	4 (2.7)	5 (1.9)	2 (0.7)	7 (1.7)	3 (0.7)	1 (1.8)	5 (5)

Myelopathy		4 (1.2)		2 (3)	6 (1)	5 (3.4)	10 (3.9)	6 (2.1)	1 (0.2)	4 (0.9)	2 (3.6)	6 (6)

Seizure Disorders	4 (9)	27 (8.3)	21 (16)	7 (11)	39 (7)	39 (26.2)	54 (20.9)	17 (6)	19 (4.5)	61 (14.3)	10 (5.5)	28 (27)

Acute Confusional State	3 (7)	12 (3.7)			15 (3)	11 (7.4)	17 (6.6)	10 (3.5)	7 (1.7)	13 (3)		7 (7)

Anxiety Disorder	6 (13)	24 (7.4)	27 (21)	4 (6)	28 (70) (*n*=40)	0	0	3 (1.1)	15 (3.6)	28 (6.6)		1 (1)

Cognitive Dysfunction	37/46 (81)	35 (10.8)	53/67 (79)	32/61 (52)	32 (52)	8 (5.4)	22 (8.5)	10 (3.5)	61 (14.6)	56 (13.1)	6 (10.9)	27 (26)
– Minor	26/37 (70)		29/67 (43)									
– Moderate	7/37 (19)		20/67 (30)									
– Severe	4/37 (11)		4/67 (6)									

Mood Disorder	20 (43)	54 (16.7)	25 (20)	17 (27)	30 (75) (*n*=40)	18 (12.1)	47 (18.1)	10 (3.5)	67 (16)	49 (11.5)		8 (8)

Psychosis		25 (7.7)	6 (5)		28 (5)	8 (5.4)	13 (5)	15 (5.3)	13 (3.1)	17 (4)	5 (9.1)	6 (6)

Acute Inflammatory Demyelinating Polyradiculoneuropathy		2 (0.6)				2 (1.3)	2 (0.8)	0	0	1 (0.2)		1 (1)

Autonomic Disorder				2 (3)	1 (0.2)	2 (1.3)	2 (0.8)	0	0	1 (0.2)		-

Mononeuropathy, Single/Multiplex			9 (7)		7 (1)	10 (6.7)	18 (6.9)	1 (0.4)	6 (1.4)	8 (1.9)		3 (3)

Myasthenia Gravis	1 (2)				1 (0.2)	0	0	2 (0.7)	2 (0.4)	2 (0.5)		-

Neuropathy, Cranial	3 (7)		2 (2)	3 (4)	8 (1.5)	11 (7.4)	11 (4.3)	7 (2.4)	14 (3.4)	9 (2)		6 (6)

Plexopathy						0	0	0	0	0		2 (2)

Polyneuropathy	13 (28)		29 (23)	8 (13)	20 (4)	8 (5.4)	10 (3.9)	0	17 (4)	16 (3.7)	4 (7.2)	2 (2)

**Figure 1:**
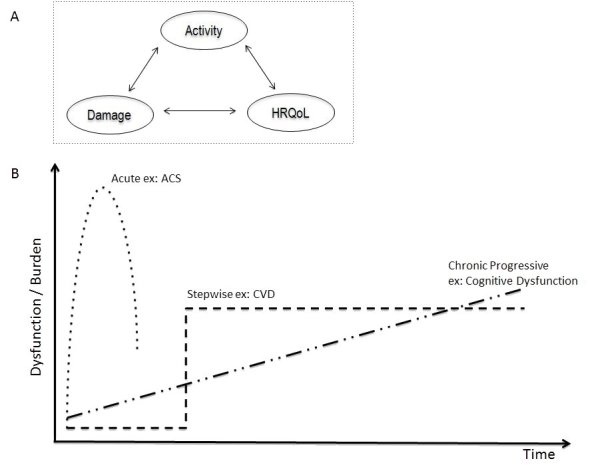
Neuropsychiatric Systemic Lupus Erythematosus (NPSLE) Manifestations Heterogeneity**.** A: The domains that should be taken into account when approaching specific or global manifestations in a patient. B: Examples of heterogeneity of a NPSLE manifestation regarding its dysfunction or burden over time. ACS, acute confusional syndrome; CVD, cerebrovascular disease; HRQoL, Health-related Quality of Life.

As SLE is a chronic disease, the global approach to patient management should take into account the following: disease activity, the accrued damage, and the functional disability as measured by health-related quality of life (HRQoL, the highest possible, Figure 1A). Each syndrome may present a different evolutionary pattern, increasing the complexity of clinical outcomes (Figure 1B).

In 1991, rheumatologists interested in SLE formed the Systemic Lupus International Collaborating Clinics (SLICC) group.^5^ The SLICC inception cohort has been collecting clinical data concerning several aspects of SLE, such as NPSLE, atherosclerosis, and damage, among others.

The latest European League Against Rheumatism (EULAR) recommendations for the management of SLE with neuropsychiatric manifestations were published in 2010 and to date remain unrevised.^6^ The clinical questions raised at the time were answered based on the literature published prior to January 2009.

In recent years, many different physiopathological pathways have been described in SLE, as well as the role of different antibodies and functional imaging techniques. What remains unknown is how these developments contribute to a better understanding of NPSLE and how they may change the way we approach our patients.

This paper provides a review of the main published papers on NPSLE since the EULAR recommendations were made, with the purpose of identifying relevant data that may potentially change current clinical practice.

## PREVALENCE

The prevalence of each NPSLE syndrome is highly variable across cohorts (Table 1),^7^–^16^ depending on patient selection method (population-based studies tend to gather less severe cases compared to tertiary center cohorts), on the nomenclature used to classify the event as NPSLE, and on whether or not the event is attributed to SLE, reaching a prevalence of almost 90%^12^ when every neuropsychiatric manifestation is attributed to NPSLE. Data from the first 572 patients in the SLICC cohort^17^ showed that 28% of individuals experienced a NPSLE event in the first 21 months of diagnosis; however, after analyzing the attribution of each event to SLE (see attribution models below), they concluded that only 6.1% was due to SLE itself.

## ARE NPSLE EVENTS DUE TO SLE ITSELF? ATTRIBUTION MODELS

When a SLE patient presents with a neuropsychiatric event, there are several concurrent causes; different attribution models have been proposed to distinguish those events, depending on whether or not they are due to SLE. In the first population-based NPSLE prevalence study, Hanna Ainiala pointed out that if the minor and most common neuropsychiatric events (headache, mild depression, anxiety, minor cognitive complaints, and electromyography-negative polyneuropathy) were excluded, then the prevalence of NPSLE would fall from 91% to 46%.^7^ These minor events became known as the “Ainiala criteria” used as exclusion events in the most relevant attribution models.^15^,^17^

The SLICC initiative NPSLE cohort led by Hanly^17^ defined factors that would indicate that the neuropsychiatric event was less likely to be related to SLE: the temporal window of development (more than 6 months before the onset of SLE); the presence of concurrent non-SLE confounders that were most likely the cause or a significant contributor to the event (as defined by the ACR nomenclature); and the “Ainiala criteria.” The use of the narrower “model B” for attribution was responsible for the drop in the early NPSLE prevalence rate from 28% (when the broader “model A” was used) to 6.1%, and the drop in the cumulative NPSLE prevalence in the 1,047 patients of the SLICC cohort from 87.6% (model A) to 24.7% (model B).^12^

Bortoluzziet al.,^15^ on behalf of The Italian Society of Rheumatology, developed a new algorithm (Table 2) that added three advantages to Hanly’s attribution models: favoring factors that supported the attribution to SLE (derived from the rationale presented in the 2010 EULAR recommendations on SLE); a list of variables of confounders and favoring factors for each of the 19 ACR NPSLE syndromes; and a scoring system that provided a positive predictive value of 86.3%, a negative predictive value of 85.7%, and a misclassification error of <10.0%. The diagnostic performance of this attribution algorithm was tested in the University of Heraklion SLE cohort, and it was superior to the other two models (i.e. Ainiala’s criteria and SLICC’s models A and B) in the major neuropsychiatric manifestations.^18^

**Table 2:** Bortoluzzi’s Attribution Algorithm, Categorization, and Weighting of Items Incorporated into the Algorithm.

	Item	Score
Item 1.	Time of the onset of NP event with respect to SLE clinical onset	
	Before (>6 months before SLE onset)	0
	Concomitant (within 6 months of SLE onset)	3
	After (>6 months of SLE event)	2

Item 2.	Minor or not specific NP events as defined by Ainiala	
	Present	0
	Absent	3

Item 3.	Confounding factors or not SLE-related associations as defined by the ACR glossary	
	None or not applicable	2
	Present (one confounding factor)	1
	Present (more than one confounding factor)	0

Item 4.	Additional (or favoring factors)	
	None or not applicable	0
	Present (one additional or favoring factor)	1
	Present (more than one additional or favoring factor)	2

ACR, American College of Rheumatology; NP, neuropsychiatric; SLE, systemic lupus erythematosus.

Like all other clinical situations, there is a need to understand the underlying cause of the neuropsychiatric event in each SLE patient in order to treat and prevent known complications and damage. The main question is: when attributing to SLE, what are we attributing to? The knowledge of SLE pathophysiology and the peculiarity of the broad mechanisms of NPSLE should be pursued to ensure the most accurate tailored approach to each patient.

## PHYSIOPATHOLOGY

**Figure 2:**
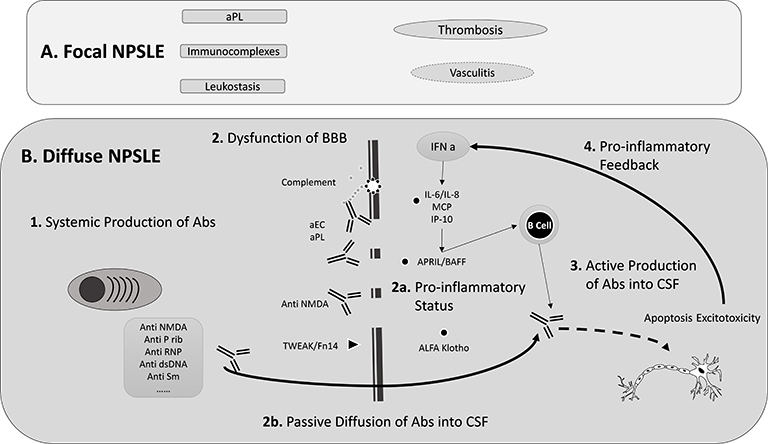
Pathophysiology of Neuropsychiatric (NP) Systemic Lupus Erythematosus (NPSLE) Focal and diffuse NPSLE differ in their pathophysiology. In the former, there is a great association with thrombotic events frequently in the context of antiphospholipid (aPL) antibodies positivity with very occasional contribution of leukostasis and vasculitis. In the latter, a more complex picture arises: systemic production of antibodies that can induce neuronal damage either directly or through induction of blood–brain barrier (BBB) dysfunction which allows passive diffusion of antibodies to the cerebrospinal fluid (CSF); activation cascade; and the development of a complex *in situ* pro-inflammatory milieu including activation of interferon α cascade. Final neurotoxicity may be mediated by induction of apoptosis, antibody-mediated damage, or excitotoxicity by receptor-agonistic binding (anti-NMDA). Abs, antibodies; anti-P rib, anti-P ribosomal antibodies; anti-Sm, anti-Smith antibodies; aEC, anti-endothelial cells antibodies; BBB, blood–brain barrier; CSF, cerebrospinal fluid.

To date, no single clear immunological pathway has been uncovered explaining why some SLE patients develop NPSLE and others do not. The main risk factors for NPSLE are general SLE activity or damage, previous NPSLE events, or other concurrent NPSLE syndromes.^6^ Although some mechanisms are common to focal and diffuse syndromes, there is a clear relationship between the presence of vasculopathy and antiphospholipid antibodies (aPL) and focal NPSLE (cerebrovascular disease, seizure, chorea, myelopathy), and between inflammatory mediators and diffuse NPSLE, but the mechanisms behind diffuse NPSLE are more elusive (Figure 2).^19^ In clear contrast to other systemic manifestations of SLE, the presence of vasculitis is not a prominent feature of NPSLE.^20^ Instead, a bland vasculopathy with rare inflammatory infiltrates is characteristic of this syndrome, with growing evidence showing that blood–brain barrier (BBB) dysfunction may be essential to the development of NPSLE, allowing the passive diffusion of auto-reactive antibodies and cytokines, facilitating the development of a pro-inflammatory milieu.^21^

### Blood–brain Barrier Dysfunction

The BBB is an essential metabolically and immunologically active barrier that controls the transport of molecules inside the central nervous system (CNS) and maintains an anti-inflammatory state. There is growing evidence supporting the presence of BBB dysfunction in NPSLE, considering the high levels of cytokines, albumin, and immunoglobulins in the cerebral spinal fluid (CSF) of these patients. Multiple insults can cause transient BBB dysfunction, such as trauma, high blood pressure, and infection.^21^ It has also been demonstrated that self-reactive autoantibodies can induce BBB dysfunction *in vitro*:^20^,^22^–^24^ anti-endothelial cell antibodies,^22^,^24^ aPL, and anti-N-methyl-D-aspartate receptor through endothelial cell binding can induce BBB disruption, allowing passive diffusion of other autoantibodies into the CSF.^20^,^23^ This interaction might induce the production of pro-inflammatory chemokines such as matrix metalloproteinase-8 (MMP-8) and plasminogen activator inhibitor-1, or cause endothelial cell dysfunction through the induction of the complement cascade.^25^

Several complement components have been associated with NPSLE: complement C3 with diffuse manifestations and complement C4 with focal NPSLE, although only with the concomitant presence of aPL.^26^ Specifically, complement C5a may contribute to BBB dysfunction by inducing the production of pro-inflammatory cytokines, promoting reactive oxygen species formation, and prompting actin reorganization.^20^

Another marker of BBB dysfunction is the development of a pro-inflammatory status detrimental to the brain tissue. The cytokine tumor necrosis factor-like weak inducer of apoptosis through its receptor fibroblast growth factor-inducible 14 has been associated with the development of BBB dysfunction in NPSLE.^21^,^27^ Soluble α klotho protein has been shown to have low levels in CSF, not only in NPSLE patients but also in other CNS inflammatory diseases (multiple sclerosis and neuromyelitis optica), suggesting a common role in neuroinflammatory modulation.^28^

Interferon α (IFNα) levels have been shown to be elevated in CSF (but not in serum) in NPSLE patients compared with SLE patients without neuropsychiatric manifestations. This elevation was correlated with the presence of interferon gamma-induced protein 10, interleukin 8 (IL-8), and monocyte chemoattractant protein-1 (MCP-1) in the CSF, cytokines/chemokines induced by IFNα and capable of inducing BBB dysfunction.^29^,^30^ Interferon gamma-induced protein 10 receptor (CXCR3) is expressed mainly in type 1 T helper cells that might suggest a preferential stimulation of this pathway; however, no studies have clarified which T helper response predominates in NPSLE.^31^ One study has also demonstrated that the antibody-antigen interaction inside the brain triggers a positive feedback loop that stimulates the production of pro-inflammatory cytokines like IFNα.^32^

### Autoantibodies

A growing number of self-reactive antibodies (in serum and CSF) have been associated with NPSLE, possibly with pathogenic roles.^25^,^33^

*Antiphospholipid antibodies* have been linked to NPSLE, especially through the induction of thrombosis causing focal manifestations of NPSLE.^19^ However, this pro-thrombotic effect cannot explain the relation of aPL with diffuse NPSLE.^34^ It has been shown that through their interaction with endothelial cells they can induce BBB dysfunction, having a synergic effect with other antibodies; additionally, they may bind neuronal cells and induce neurotoxicity.^22^

*Anti-dsDNA antibodies*, a specific marker of SLE activity, have been detected in CSF of NPSLE patients and proven to bind hippocampal neurons.^35^ A subset of anti-dsDNA antibodies have been shown to interact with a subunit of anti-N-methyl-D-aspartate and its receptor; these complexes have been related to mood disorders, acute confusional state (when in high titers), and cognitive decline including memory dysfunction.^20^,^36^ These antibodies may induce neuronal death by complement cascade activation and induce cellular calcium overload and neurotoxicity similar to that occurring in Alzheimer’s disease. In mice it has been demonstrated that this action can be blocked by an anti-N-methyl-D-aspartate receptor antagonist (memantine).^37^ However, in a study of SLE patients with cognitive dysfunction, those in treatment with memantine did not have better cognitive outcomes.^25^

The cross-reactivity of dsDNA with anti-N-methyl-D-aspartate receptor is an example of how antigenic mimicry may contribute to the pathogenesis of autoimmune diseases. Another example is the cross-reactive binding of the *anti-ribosomal P protein* with neuronal surface antigens (neuronal growth-associated protein 43).^22^ These antibodies have been shown to interact with endothelial cells, and, in particular, they specifically target the hippocampus and the amygdala, which possibly explains its association with depression and cognitive dysfunction. However, there has been contradictory evidence linking anti-ribosomal P protein to NPSLE manifestations.^22^,^29^ *Anti-Smith antigen antibodies* (anti-Sm) have been shown to cross-react with anti-ribosomal P protein and are specifically associated with acute confusional state.^36^

*Anti-ribonucleoprotein* (RNP1) titers in CSF have been associated with the presence of neuropsychiatric manifestations in both SLE and mixed connective tissue disease.^38^ Anti-aquaporin 4 is a marker of neuromyelitis optica (Devic’s disease), a multiple sclerosis-like disease that can occur on its own or be a manifestation of NPSLE.^23^ Anti-glyceraldehyde 3-phosphate dehydrogenase was found to be a marker of schizophrenia and depression both in patients with and without SLE and to directly induce dendritic atrophy and cognitive and emotional dysfunction in mice after intrathecal injection.^39^

*Anti-SSA* and *anti-P ribosomal antibodies* were found in CSF of NPSLE patients and correlated with NPSLE manifestations, compared to SLE patients with no neuropsychiatric manifestations.^40^ Anti-microtubule-associated protein-2, anti-glial fibrillary acid protein, anti-C1q, anti-lymphocytotoxic antibodies, and anti-ganglioside antibodies have been inconsistently associated with various NPSLE syndromes.^22^,^25^,^29^,^41^

#### Systemic versus Local Production of Antibodies

The apparent role of antibodies in NPSLE raises the question as to whether they are produced systemically and passively diffused to the CSF through a dysfunctional BBB, or if they are locally produced inside the CNS. The association of high CSF antibodies titers (anti-Sm and anti-ribosomal P protein) with increased levels of CSF albumin, together with the evidence of BBB dysfunction, supports the hypothesis of passive diffusion.^36^ On the other hand, a study with anti-N-methyl-D-aspartate receptor has supported the role of locally produced antibodies as suggested by the increased ratio of CSF/serum antibodies.^42^ Other studies have demonstrated an association between high CSF levels of B cell surviving factors (a proliferation-inducing ligand (APRIL) and B cell-activating factor (BAFF)) and NPSLE manifestations that may support local production of antibodies by B cells.^43^

### Genes and NPSLE Risk

Genetic studies are very difficult in heterogeneous diseases such as SLE. Nevertheless, some insights have come to light in the past couple of years. The discovery of the TREX1 gene (IFN regulator gene), responsible for Aicardi–Goutières syndrome, led to the recognition of association between several polymorphisms in heterozygotes with high NPSLE risk.^44^ The TRPC6 gene, involved in the regulation of N-methyl-D-aspartate receptor, seems to be protective of NPSLE manifestations.^45^

## NPSLE: SCREENING, DIAGNOSIS AND MONITORING

In clinical practice, NPSLE is usually diagnosed when the patient is symptomatic (based on patient complaints or the physician’s assessment). In clinical trials and inception cohorts, several screening protocols have been used, including general queries, mood disorders, and cognitive dysfunction scales, which are time-consuming and difficult to use in routine clinical practice. Mosca et al. developed a simple self-assessment screening tool for NPSLE in routine clinical practice with 27 questions for the 19 ACR NPSLE syndromes that capture sub-clinical involvement.^46^ A score above 17 was considered as suggestive of the presence of NPSLE with a sensitivity of 92.9% (95% CI 85.1%–97.3%) and specificity of 25.4% (95% CI 14.7%–39.00%). Efforts have been made to validate it in different countries.

The initial diagnosis workup for NPSLE manifestation is the same as for the same syndrome in non-SLE patients and depends upon the type of neuropsychiatric manifestation. The complementary exams are done mainly for exclusion of other diagnoses, as none of the results are specific of NPSLE.^6^ The neuropsychological assessment of cognitive function should be done not only for diagnosis but also to have a neuropsychiatric baseline for monitoring the patient’s status over time.

When EULAR recommendations for NPSLE management^6^ were compared with the usual care in a tertiary rheumatology center, an overutilization of magnetic resonance imaging (MRI) and suboptimal evaluation of cognitive dysfunction were noted in clinical practice as compared to the recommendations.^47^

## IMAGING TECHNIQUES IN NPSLE

Magnetic resonance imaging (MRI) has become the gold standard tool for NPSLE assessment, replacing computed tomography (CT) in the evaluation of brain pathology among these patients. Despite its low specificity for neuroradiological findings in NPSLE, MRI has proved to have a sufficient level of accuracy and has the advantage of allowing the exclusion of other neurological conditions. The average sensitivity of MRI in active NPSLE is 57%, and the recommended MRI protocol (brain and spinal cord) includes conventional MRI sequences—T1/T2, fluid-attenuated inversion recovery, diffusion-weighted imaging, and gadolinium-enhanced T1 sequence.^6^ A wide variety of conventional MRI findings have been previously described, albeit more than half of patients with NPSLE present a normal MRI.^48^

The MRI changes described in NPSLE patients range from small punctate focal lesions in white matter, defined as white matter hyperintensities, to severe large lesions. Abnormal findings may be divided in three groups, according to their pathophysiology and imaging features: small-vessel disease, large-vessel disease, and inflammatory-like lesions.^49^

Small vessel disease, which accounts for 30%–75% of MRI findings in NPSLE, includes white matter hyperintensities, cortical brain atrophy, lacunes, small subcortical infarcts, and microbleeds,^50^,^51^ with white matter hyperintensities being the most commonly documented in SLE patients. The majority of small vessel lesions are often considered non-specific, as they may be related to age, hypertension, disease duration, low complement, aPL antibodies, and the presence of NPSLE manifestations, mainly cognitive dysfunction, seizures, and cerebrovascular disease.^52^ Given their non-specificity, part of the white matter hyperintensities observed in patients with active NPSLE do not seem to be related to SLE and are not responsible for neurological symptoms, while a significant percent of these lesions are transitory and disappear with time and immunosuppressive therapy.^48^ In patients with a more severe disease, white matter hyperintensities may be a consequence of central nervous system damage and progress over time.^51^ Signs of cortical atrophy in the MRI usually occur concurrently with other small vessel lesions.^52^ Cortical atrophy seems to correlate with disease duration, cognitive dysfunction, cerebrovascular disease, seizure,^53^ low complement, and lupus anticoagulant; some studies also found a significant positive association with corticosteroid therapy.^48^,^52^

Large vessel disease is considerably less frequent than small vessel lesions and accounts for 10%–15% of NPSLE MRI findings.^48^ Large vessel disease causes medium to large size vessel infarcts, which can be single or multiple, extending through a vascular territory, and involving both the grey and white matter.^51^

Inflammatory-like lesions are rather less common, accounting to for 5%–10% of NPSLE.^54^ Myelitis is a rare manifestation of NPSLE that affects 1%–5% of SLE patients, despite being a serious complication of the disease with a variable outcome. Contrast-enhanced spinal cord MRI also helps to exclude cord compression, and brain MRI is recommended when other NPSLE symptoms or signs coexist, as well as in the differential diagnosis of demyelinating disorders. Moreover, when there is a suspected cranial neuropathy, MRI is crucial for evaluation of optic nerve enhancement and brain abnormalities.

### Functional Imaging as a Tool to Pursue NPSLE Mechanisms

Despite the utility of conventional MRI for evaluation of neurologic symptoms of SLE patients, the lack of sensitivity of this neuroimaging method has led to the exploration of other techniques. More advanced methods of neuroimaging might be performed when conventional MRI does not contribute to explain neurologic signs and symptoms in SLE patients and are also useful to distinguish between acute and chronic lesions. These neuroimaging techniques include quantitative MRI, such as magnetic resonance spectroscopy, magnetization transfer imaging, diffusion tensor imaging, and diffusion-weighted imaging, or fluorodeoxyglucose positron emission tomography (FDG-PET) and single photon emission CT (SPECT).

Magnetic resonance spectroscopy is a recent modality that can non-invasively quantify some brain biochemical compounds such as reduction in N-acetyl aspartate representing neuronal or axonal dysfunction or loss.^55^ Reduction in N-acetyl aspartate seems to correlate with cerebral atrophy and neurocognitive dysfunction, especially in SLE patients with aPL antibodies,^55^,^56^ but no strong correlation with global disease activity has been described so far.

Diffusion tensor imaging and diffusion-weighted imaging are non-invasive MRI techniques that measure the diffusion of water in the brain, assessing the white matter structure and integrity. Diffusion-weighted imaging has shown abnormal diffusivity consistent with inflammation and loss of white matter integrity; it also allowed discernment between inflammatory and ischemic lesions in SLE patients. Nevertheless, this technique does not contribute significantly to the distinction from other causes of ischemic or inflammatory lesions.^57^ Magnetization transfer imaging quantifies the exchange of protons between those bound in macromolecules, like cholesterol in myelin, and free water. However, the application of these techniques to NPSLE is still quite limited.

Several studies have demonstrated FDG-PET hypometabolism in at least one brain region of patients with major or minor CNS symptoms, mainly in parietal, temporal, fronto-temporal, and central regions.^58^ These findings are particularly important for patients with depressive symptoms related to NPSLE, since in these cases brain hypometabolism is preferentially detected in the parieto-occipital region, while in patients with primary depression the location of hypometabolic lesions is commonly found in bilateral insula and basal ganglia.^57^ Some authors have also described an improvement of clinical and FDG-PET findings with immunosuppressive therapy among these patients.^58^,^59^

The SPECT modality provides an estimate of regional cerebral blood flow, metabolism, and neuronal activity and is extremely sensitive in the detection of abnormalities. Among NPSLE patients SPECT has identified diffuse and focal deficits, but they are not specific and do not always correlate with neuropsychiatric manifestations. The most notable SPECT abnormality in patients with major NPSLE is patchy diffuse hypoperfusion, mainly associated with acute major NPSLE events, whereas focal lesions appear to be more common in patients with longer duration of disease. Both FDG-PET and SPECT have been explored as functional imaging tools in lupus and appear to be sensitive in detecting subtle brain changes in NPSLE. Nonetheless, both techniques, especially SPECT, have low specificity, which limits their use in clinical practice.^55^

Considering the multifactorial basis of brain disease in SLE patients, its diagnosis remains a challenge. Therefore, a multimodal approach, including a combination of both morphological and functional imaging, may improve the current knowledge of metabolic and structural changes related to NPSLE syndromes.

## MANAGEMENT

**Figure 3:**
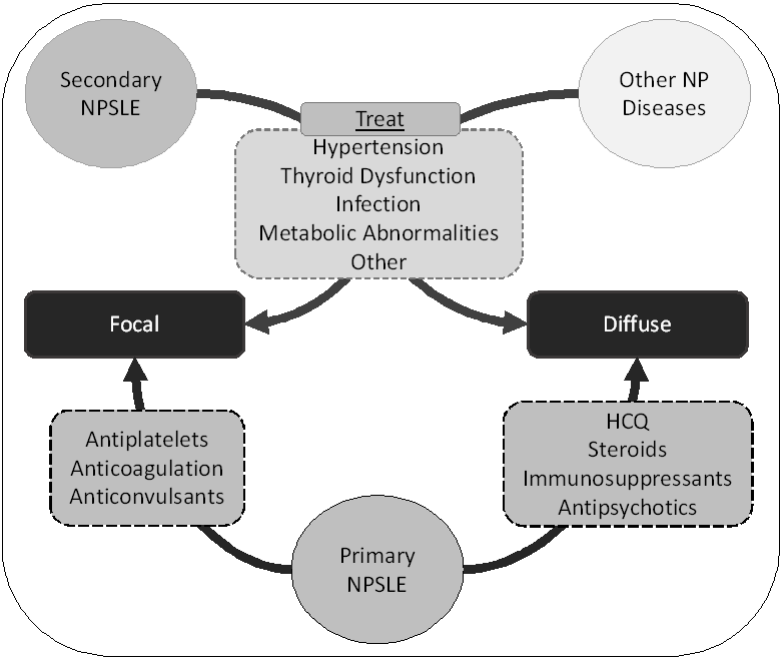
Treatment Approach to Neuropsychiatric (NP) Systemic Lupus Erythematosus (NPSLE) Diffuse or focal NP presentations may arise (1) directly as a result of autoimmune mechanisms (primary NPSLE), in which case treatment should target the most relevant pathologic mechanism (anticoagulation in aPL-associated thrombosis or immunosuppression in antibody-mediated diffuse damage); (2) secondary to complications of the disease or treatment (secondary NPSLE), which may require treatment such as antibiotic therapy for bacterial infection; and (3) finally as a consequence of NP events related to SLE, which may have specific treatment. HCQ, hydroxychloroquine.

The management of NPSLE must be directed at the type of neuropsychiatric event (among the 19 NPSLE syndromes) and its characteristics: acute versus chronic; predominantly vascular versus inflammatory; minor versus severe; active and ongoing versus damage (Figure 3).

Very few studies have been conducted assessing the role of drugs in the prevention of NPSLE events. In the LUMINA cohort,^60^ hydroxychloroquine and moderate prednisolone dose delayed the first NPSLE manifestation, regardless of the type of event. The SALUD study^61^ showed that aspirin improved cognitive function in older patients with risk factors. In our cohort,^62^ the odds of having cognitive impairment was significantly lower for patients taking hydroxychloroquine (adjusted odds ratio 0.368; *P*=0.036) even when controlling for other covariates (i.e. education, disease duration, and neuropsychiatric involvement), and among patients without NPSLE (*n*=96) the odds of having cognitive impairment remained significantly lower for patients taking hydroxychloroquine when controlled for other covariates (i.e. education, disease duration, anxiety, and depression).

When the neuropsychiatric event is acute and diffuse, it is presumably mainly inflammatory and almost always associated with generalized SLE activity. In this scenario, SLE global activity should be controlled at the same time as NPSLE is assessed,^6^ and if NPSLE is severe (acute confusional state, seizures, encephalitis) it should be treated with immunosuppressive drugs. There are no strong data supporting one or another immunosuppressor. Steroids have been used in several different doses, from prednisolone 0.5–1 mg/kg/day to bolus of endovenous methylprednisolone 3–5 days of 500 mg to 1 g/day.^63^,^64^ There are several case series, open label studies, and one observational trial supporting the use of cyclophosphamide,^65^ and the most described regimens are the low-dose EuroLupus regimen endovenous 500 mg every other week for six cycles and the USA National Institutes of Health regimen of 1 g/monthly for 6 months. A Cochrane Review showed that there is very little evidence that cyclophosphamide has better outcomes than methylprednisolone alone.^66^ There are case series reporting the use of azathioprine in maintenance treatment after cyclophosphamide or high-dose steroids in severe NPSLE,^64^ and a few case reports of patients that were successfully treated with mycophenolate mofetil.^64^,^67^ A recent revision summarized the positive effects of rituximab (with high rates of response) and belimumab in severe NPSLE.^68^ In severe refractory cases, intravenous immunoglobulin^69^,^70^ and plasmapheresis^71^,^72^ have been successfully used as bridge therapy, mainly when infection is not completely ruled out, in pregnant patients, or when there are life-threatening symptoms.

If the patient has a focal neuropsychiatric syndrome, there is a high probability of aPL positivity. In these cases, ischemic and cerebral venous thrombotic events should be anticoagulated.^73^ Other focal syndromes (seizures, chorea, and transverse myelopathy) are also patterns of microvascular disease, and they might benefit from antiplatelet drugs or anticoagulation. If there are other clinical or laboratorial signs of inflammation, additional immunosuppression should be considered. The most suitable management for these patients should be decided case by case. Despite these recommendations, not every NPSLE manifestation requires immunosuppressive treatment. When Pamfil et al. audited the usual care in NPSLE against EULAR recommendations in two tertiary centers,^47^ they found that 52% of cerebrovascular events were overtreated with immunosuppressive therapy in addition to antithrombotic treatment.^47^

Symptomatic adjuvant therapy is generally needed to control either severe (acute confusional syndromes, psychosis, seizures, chorea) or minor neuropsychiatric syndromes (anxiety, mood disorders, headache) (Figure 3).^6^ These patients should be managed by a multidisciplinary team led by a rheumatologist/internal medicine expert and including a neurologist, psychiatrist, psychologist, and physiotherapist.

## CONCLUSIONS

With the growing knowledge and recognition of different heterogenic syndromes under the shared title of NPSLE there is an unmet need for reclassification of NPSLE patients according to both clinical phenotypes and immunological profile. As such, the NPSLE attribution might change from the disease (SLE or not) to the related pathophysiology: anti-aquaporin-4 attribution, anti-N-methyl-D-aspartate receptor attribution, aPL attribution, anti-P ribosome attribution, anti-SSA attribution, etc. The holistic approach to each patient with NPSLE should be commenced, taking into account patients’ SLE-related conditions and comorbidities, the probable role of SLE physiopathology, and the need for specific treatment.

In the future, and as more data become available, we will hopefully be able to tailor the approach and management of NPSLE based on the clinical presentation, immunological phenotype, and genotype pattern.

## Abbreviations

ACR: American College of Rheumatology
aPL: antiphospholipid antibodies
APS: antiphospholipid antibodies syndrome
BAFF: B cell-activating factor
BBB: blood–brain barrier
HRQoL: health-related quality of life
CNS: central nervous system
CSF: cerebrospinal fluid
CT: computed tomography
dsDNA: double strand DNA
EULAR: European League Against Rheumatism
FDG-PET: fluorodeoxyglucose positron emission tomography
HCQ: hydroxychloroquine
IFNα: interferon-α
IL-8: interleukin 8
IP-10: interferon gamma-induced protein 10
GAPDH: glyceraldehyde 3-phosphate dehydrogenase
MCP-1: monocyte chemoattractant protein-1
MMP-8: matrix metalloproteinase-8
MRI: magnetic resonance imaging
NPSLE: neuropsychiatric systemic lupus erythematosus
RNP1: ribonucleoprotein 1
SLE: systemic lupus erythematosus
SLICC: Systemic Lupus International Collaborating Clinics
Sm: Smith
SPECT: single photon emission computed tomography
